# Correlation between a motion analysis method and Global Operative Assessment of Laparoscopic Skills for assessing interns’ performance in a simulated peg transfer task in Jordan: a validation study

**DOI:** 10.3352/jeehp.2025.22.10

**Published:** 2025-03-06

**Authors:** Esraa Saleh Abdelall, Shadi Mohammad Hamouri, Abdallah Fawaz Al Dwairi, Omar Mefleh Al-Araidah

**Affiliations:** 1 Industrial Engineering Department, Jordan University of Science and Technology, Irbid, Jordan; 2 Faculty of Medicine, Al-Balqa Applied University, Alsalt, Jordan; 3 Faculty of Medicine, Jordan University of Science and Technology, Irbid, Jordan; 4 King Abdullah University Hospital, Irbid, Jordan; The Catholic University of Korea, Korea

**Keywords:** Internship and residency, Laparoscopy, Minimally invasive surgical procedures, Surgeons, Jordan

## Abstract

**Purpose:**

This study aims to validate the use of ProAnalyst (Xcitex Inc.), a software for professional motion analysts to assess the performance of surgical interns while performing the peg transfer task in a simulator box for safe practice in real minimally invasive surgery.

**Methods:**

A correlation study was conducted in a multidisciplinary skills simulation lab at the Faculty of Medicine, Jordan University of Science and Technology from October 2019 to February 2020. Forty-one interns (i.e., novices and intermediates) were recruited and an expert surgeon participated as a reference benchmark. Videos of participants’ performance were analyzed through the ProAnalyst and Global Operative Assessment of Laparoscopic Skills (GOALS). Two results were s analyzed for correlation.

**Results:**

The motion analysis scores by Proanalyst were correlated with those by GOALS for novices (r=–0.62925, P=0.009), and Intermediates (r= –0.53422, P=0.033). Both assessment methods differentiated the participants’ performance based on their experience level.

**Conclusion:**

The motion analysis scoring method with Proanalyst provides an objective, time-efficient, and reproducible assessment of interns’ performance, and comparable to GOALS. It may require initial training and set-up; however, it eliminates the need for expert surgeon judgment.

## Graphical abstract

[Fig f8-jeehp-22-10]

## Introduction

### Background/rationale

Minimally invasive surgery training programs are continuously evolving, with a trend toward using validated simulation‐based models [1-4]. Among these, the Fundamentals of Laparoscopic Surgery program was developed to target essential minimally invasive surgery skills [5,6]. In addition, various assessment methods have been used to evaluate surgical skills. For instance, the Objective Structured Assessment of Technical Skills [6], Global Operative Assessment of Laparoscopic Skills (GOALS) [6,7], and Operative Performance Rating System [8] all use global rating scales to assess surgical skills. Despite their ability to assess minimally invasive surgery skills, these tools are highly dependent on the availability and judgment of senior surgeons, limiting their generalizability and objectivity [9]. Other methods, such as the Observational Clinical Human Reliability Assessment and Generic Error Rating Tool, relied on recorded videos of procedures to count errors for performance assessment. Therefore, there was a need for an objective and time‐efficient assessment method to evaluate surgical trainees’ skills, deliver timely feedback, and monitor their progress. Hence, there has been a shift toward using virtual reality (VR) and augmented reality (AR) simulators to objectively assess performance and increase training fidelity; however, these simulators are expensive and have issues with accessibility and comprehensiveness [10-13]. An alternative approach is to use motion analysis to objectively quantify trainees’ minimally invasive surgery skills. Different motion tracking systems have been explored, ranging from electromagnetic, optical, and infrared systems to video-based computer software. These systems tracked hand movements during procedures, using markers, cameras, and other tools to capture motion variables. The captured variables commonly included task time, path length, velocity, acceleration, jerk, force, and motion smoothness [14]. Notably, most of these motion analysis methods focused on intracorporeal suturing and knotting performed on animal models, with few being applied in simulators. However, to our knowledge, other basic minimally invasive surgery skills—such as dexterity, depth perception, and eye–hand coordination—have not been fully examined using motion tracking. Summary table of additional referencing reinforcing research was presented in Supplement 1.

### Objectives

We introduced Proanlyst (Xcitex Inc.), motion tracking analysis software within a minimally invasive surgery simulator to analyze performance and technical surgical skills. Furthermore, the ismotion tracking and analysis method was compared with GOALS. Additionally, we tested the capability of the former technique in differentiating the motion characteristics of intermediates and novices, and compared them to an expert as a reference benchmark while performing a simulated peg transfer task

## Methods

### Ethics statement

This research was approved by the institutional review board at Jordan University of Science and Technology (IRB approval number: 20190327). Informed consent was obtained from all participants.

### Study design

It is a validation study of ProAnalyst (Xcitex Inc.), a software for professional motion analysts for assessing interns’ performance of the minimally invasive surgery simulation task. An experienced surgeon’s performance was also assessed as a reference point to contextualize the results, rather than as a direct comparison group.

### Setting

This study was done between October 2019 and February 2020 in a multidisciplinary skills simulation lab at Faculty of Medicine, Jordan University of Science and Technology.

#### Minimally invasive surgery simulated tasks

**Fig. 1.**
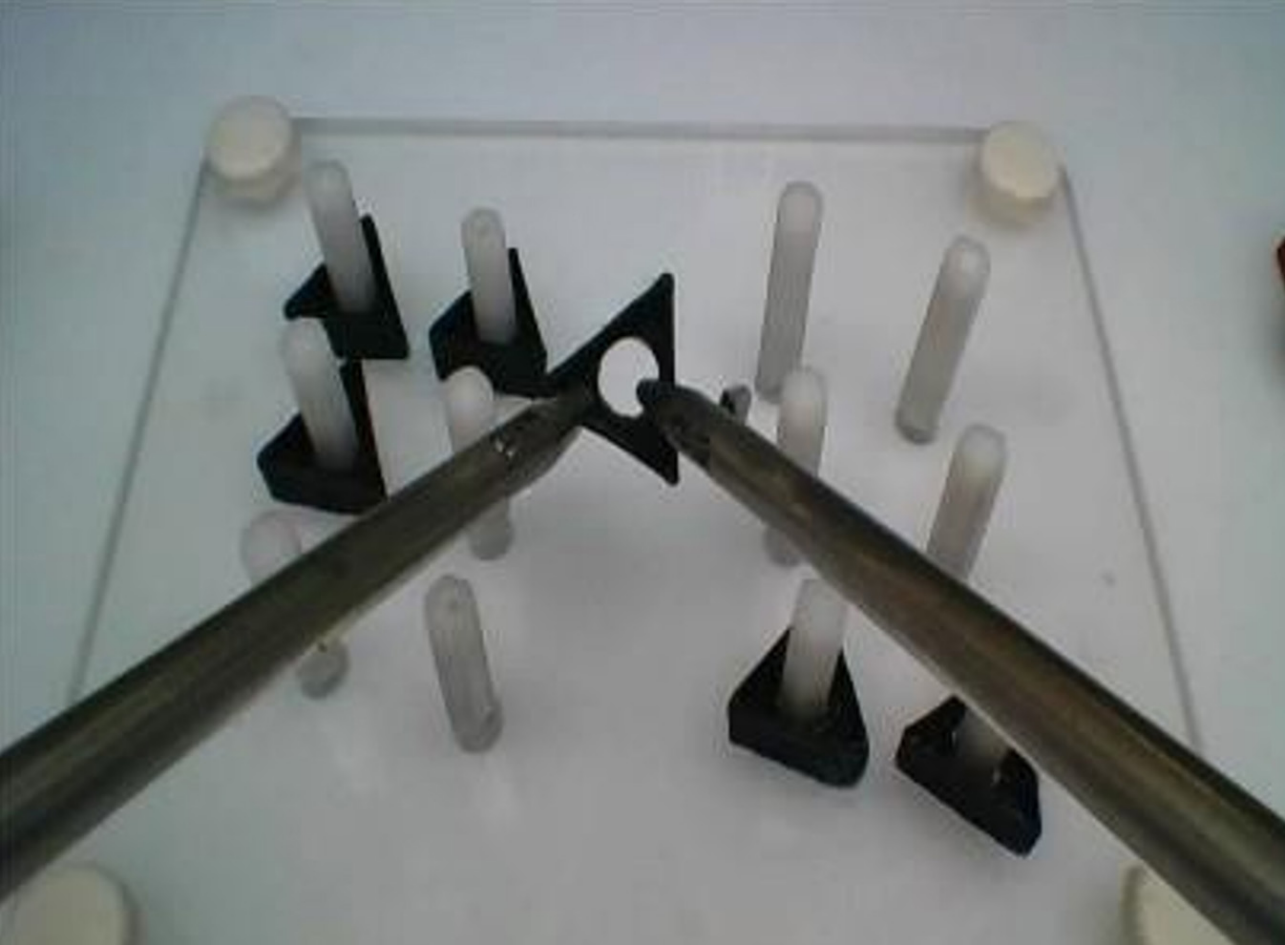
Peg transfer task with a 3-dimensional–printed model.

The peg transfer task was selected for participants because it is not technically challenging and is therefore suitable for novices [14] (Fig. 1). The peg transfer task consists of 6 plastic triangles that are transferred from one side of a pegboard to the other using laparoscopic graspers. An error in performing the peg transfer task was defined as any dropped triangle. In this study, a modified version of the traditional peg transfer task was used, as detailed in the next section.

#### Development phase and pilot study

A modified peg transfer task with uniform coloring was developed. The triangular pegs of the peg transfer task were 3- dimensional printed from plexiglass, using the exact standard dimensions of pegs in Fundamentals of Laparoscopic Surgery. All pegs had the same color (i.e., black) to clearly capture the effect of participants’ experience level on performance, without any overlap resulting from different peg colors, as seen in the traditional peg transfer task. The decision on the colors was reached after a small-group discussion with a multidisciplinary team of subject matter experts (SMEs). The SME team included a human factors and ergonomics engineer, a biomedical engineer, a thoracic surgeon, and an minimally invasive surgery surgeon with experience in surgical residency education and training. Once the design for the new peg transfer task was finalized, it was tested with 9 novice interns to refine task elements and timing and to standardize performance measures.

#### Procedure

The experiment was conducted in a closed room at a multidisciplinary skills simulation lab. At the beginning, novice participants filled out a demographic survey, then they received a training session on how to perform the peg transfer task. After the training session, the experiment was conducted, during which each novice participant performed the task 3 times. The expert as well as the intermediates were asked to perform 3 trials of the task after receiving the same training session. The task was performed in a box trainer, and laparoscopic graspers were used. A 1080 HD camera was used to both display and record the scene from the enclosed simulation box on a monitor. All trials were video-recorded for subsequent analysis using Proanaylst software. Raters assessed the subjects performance using GOALS during and after the trials. The post-experiment assessment was adopted to allow reviewing the recorded videos multiple times for more accurate assessment of subjects’ performance.

### Participants

A total of 41 surgical interns were recruited for this research. Nine of them participated in a pilot study to refine the experimental setup and were not included in the final analysis. The remaining 32 surgical interns (16 novices and 16 intermediates) participated in the actual study. In addition, an expert surgeon with experience in performing more than 30 minimally invasive surgery procedures was recruited as a reference benchmark. Participation was entirely voluntary, and participants were allowed to quit at any time without penalty.

### Variables

Outcome variables were interns’ performance of a simulated peg transfer task listed in Table 1.

### Data sources/measurement

The performance was evaluated by tracking the motion of the surgical grasper with ProAnalyst, motion analysis method as well as by using GOALS.

#### Motion tracking, data extraction, and parameters

The recorded videos of participants’ trials were imported into ProAnalyst (Xcitex) motion analysis software. The motion of the surgical tool tips was captured as movement in both the x and y axes while performing the task. The motion data (Supplements 2, 3) were then analyzed and modeled in R (Supplement 4) to extract the motion parameters, as defined in Table 1. A score out of 5 was derived for each parameter in Table 1 and used to calculate a motion analysis composite score out of 5. The expert video was also analyzed in a similar manner to serve as a reference against which the participants’ performance was compared.

#### GOALS assessment and scoring

Herein, we only considered the dexterity, depth perception, autonomy, efficiency, and operation flow metrics of GOALS for assessing the performance of participants, as they reflect the selected peg transfer task task, as recommended by [12] (Table 1). Two raters were trained and instructed to use GOALS during the pilot study.

Two expert raters were trained and instructed to use GOALS during the pilot study. The raters were then asked to blindly and independently evaluate the participants’ video recordings. They assessed each of the aforementioned metrics on a scale from 1 to 5, depending on the participants’ performance. Subsequently, Spearman correlation was used to test the consistency of their ratings. Spearman correlation for expert raters’ scores showed that inter-rater reliability scores ranged between 0.79 and 0.92. The correlation test results suggest consistency in the raters’ scoring and reflect that the experiment was performed within a controlled test environment.

### Bias

There was no selection bias since all target subjects were included.

### Study size

Since all target subjects were included, there was no sample size estimation.

### Statistical methods

Analysis of variance was used to compare the performance of novices and intermediates. The expert’s data were not included in the statistical comparisons but were used as a reference to contextualize performance differences. Data were analyzed using IBM SPSS ver. 19.0 (IBM Corp.) with a significance level of 0.05. The correlation between participants’ motion analysis scores and GOALS scores, as well as between the scores assigned by 2 raters (inter-rater reliability), was examined using Spearman’s rank correlation coefficient (Supplements 5–7).

## Results

### Participants

Descriptive statistics can be found in Table 2. It is worth noting that none of the novices had prior experience with Fundamentals of Laparoscopic Surgery–peg transfer task, unlike the intermediates, who were trained for 1 month on Fundamentals of Laparoscopic Surgery–peg transfer task. More men participated in this study; however, they were counterbalanced across the groups.

### Main results

#### Measurement of performance with motion analysis method

**Fig. 2.**
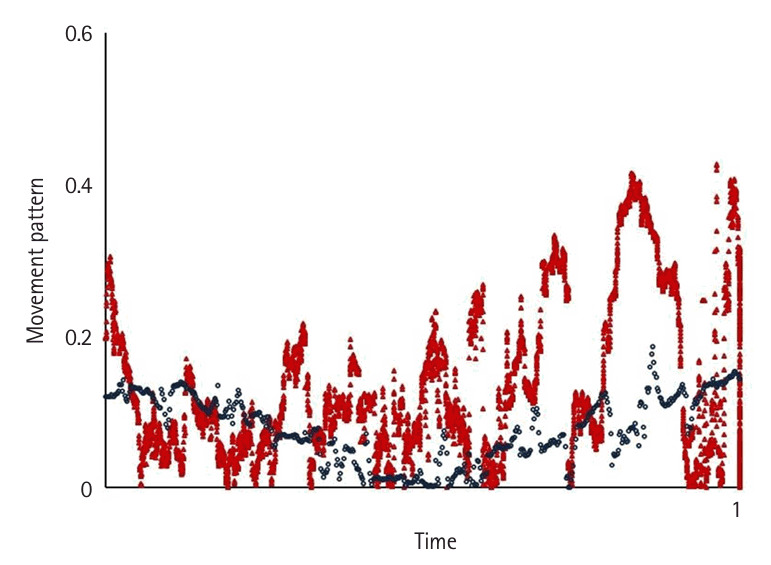
Comparison of the average movement pattern between intermediates (red) and the expert (blue) as the reference benchmark while performing peg transfer task (Dataset 1).

**Fig. 3.**
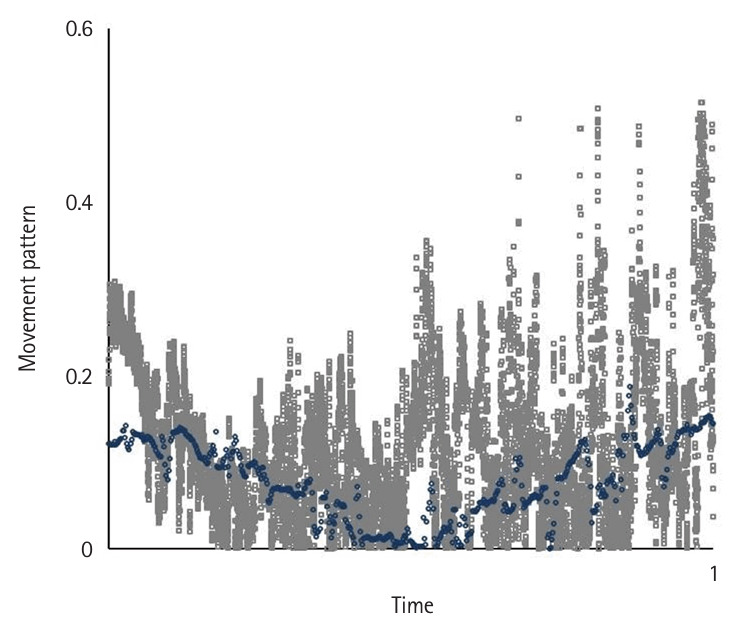
Comparison of the average movement pattern for novices (gray) and the expert (blue) as the reference benchmark while performing peg transfer task (Dataset 1).

Motion variables (i.e., task time, movement pattern, tremor, and extreme movement) differed significantly according to the level of participants’ experience (P<0.05) (Figs. 2, 3). Novices spent significantly more time—an average of 7.53±3.9 minutes—completing the tasks compared to intermediates, who took 2.29±1.11 minutes on average (P<0.05), and showed a higher number of movement errors, with an average of 3.66 triangle drops, compared to intermediates, who dropped 0.75 triangles on average.

**Fig. 4.**
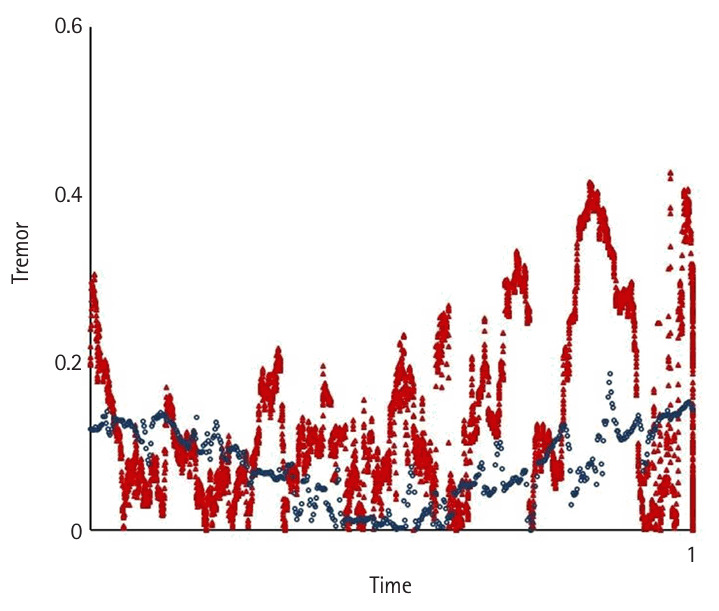
Comparison of the average hand tremor between intermediates (red) and the expert (blue) as a reference benchmark while performing peg transfer task (Dataset 1).

**Fig. 5.**
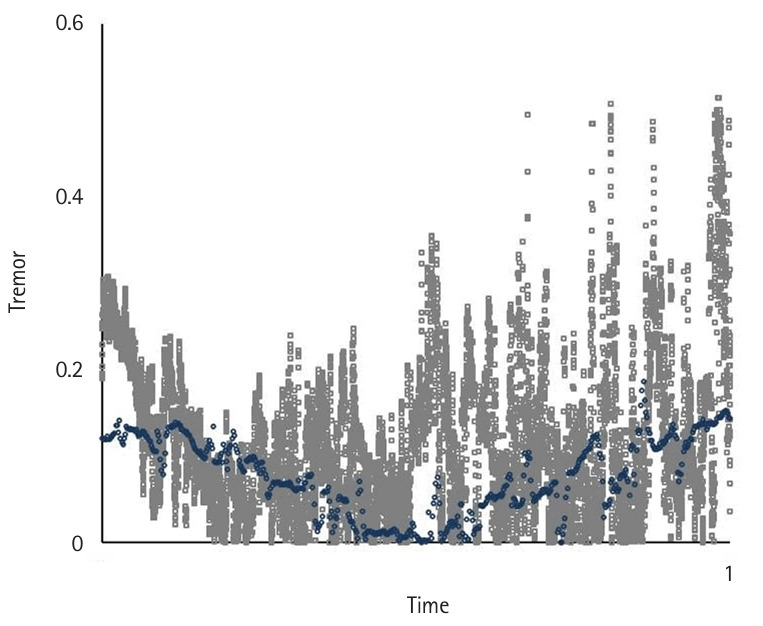
Comparison of the average hand tremor between novices (gray) and the expert (blue) as the reference benchmark while performing peg transfer task (Dataset 1).

**Fig. 6.**
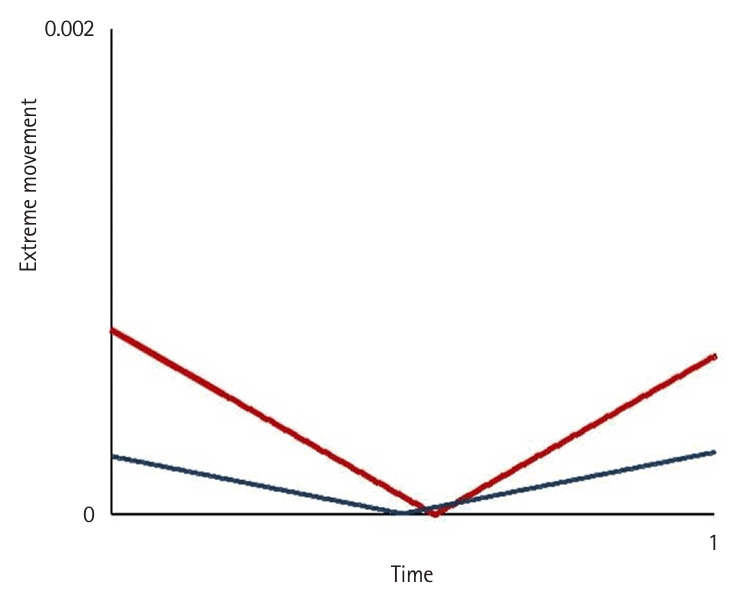
Comparison of the average extreme movement between intermediates (gray) and the expert (blue) as a reference benchmark while performing peg transfer task (Dataset 1).

**Fig. 7.**
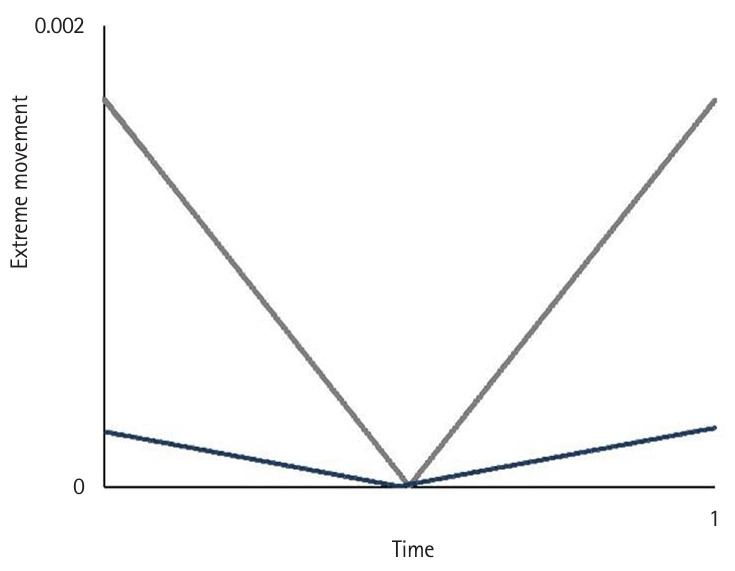
Comparison of the average extreme movement between novices (gray) and the expert (blue) as a reference benchmark while performing peg transfer task (Dataset 1).

Novices had the worst movement pattern, tremor, and extreme movements, followed by intermediates. The expert’s performance was analyzed separately as a reference benchmark, demonstrating a substantially faster completion time and a more efficient movement pattern. In the expert, performance was characterized by a lack of wasted moves and an appropriate pace while executing the task using fluid, concise movements (Figs. 2–7, Supplement 5).

#### Measurement of performance with GOALS

The level of participants’ experience significantly affected bimanual dexterity, depth perception, autonomy, efficiency, and operation flow (P<0.05) (Supplement 6). Novices scored the lowest GOALS composite score of 1.35, followed by intermediates, who scored 2.95, compared to the expert reference, who scored 4.55.

#### Correlation of performance with between motion analysis and GOALS

Motion analysis composite scores correlated well with those by GOALS for novices (r=–0.62925, P=0.009), and intermediates (r= –0.53422, P=0.033) (Supplement 7). The negative correlation implies that as goal score increases, the motion analysis score decreases (i.e., tremor, extreme movement, errors,etc.).

## Discussion

### Key results

The current study supported the validity of the Proanalyst, motion analysis scoring method by correlating it with traditional GOALS scoring in assessing laparoscopic performance of interns. Furthermore, all participants were exposed to the same training on the peg transfer task, during which the motion of the surgical instruments (i.e., graspers) was analyzed and modeled mathematically, resulting in reproducible and objective outcomes. The motion analysis scoring method effectively differentiated novices from intermediates. The study showed that the motion analysis method is more time efficient, reliable, standardized, reproducible and eliminate the biases and subjectivity issues existed in GOALS scoring.

### Interpretation

The results demonstrated that both scoring methods were able to differentiate novices from intermediates. The GOALS composite score, as well as the motion analysis score, was lowest for novices, and followed by intermediates. Examination of the motion analysis variables revealed that the performance of both novices and intermediates was characterized by the graspers hovering before, during, and after picking up and placing the triangular pieces. In fact, novices required an adaptation period before they could perform the task correctly and cleanly. This is supported by the better performance of intermediates, who had prior experience with the task for 1 month.

### Comparison with previous studies

Similar to the findings of McGoldrick et al. [5], the GOALS composite score correlated well with the motion analysis score and was significantly higher. This may be attributed to the subjectivity in the judgment of raters compared to motion analysis scoring. Human judgment is susceptible to random error as well as leniency bias, which can result in an overestimation of participants’ performance, thereby explaining the higher GOALS scores. Another reason for higher subjective scores is the so-called halo effect, in which a positive impression can influence scoring, or social desirability bias, where judges tend to give scores that are expected. On the other hand, even with rater training, the lack of a predefined and strict protocol for the subjective assessment method can exacerbate unconscious bias, leading to overestimation of participants’ performance and variation in how the rating guidelines are understood and applied. Other factors that can affect subjective assessments include contextual factors such as the judge’s mood, recent experiences, and environment. In contrast, motion analysis scoring methods involve the use of technology, analysis software, and mathematical modeling, resulting in more reliable, well-defined, standardized, and reproducible scores that eliminate the biases and factors discussed earlier.

Furthermore, the findings of this work showed that the 2 scoring methods are capable of differentiating participants’ performance scores. These results were consistent with the findings of McGoldrick et al. [5], where motion analysis of a surgeon’s hand during microsurgery differentiated experts from novices. Other studies demonstrated that motion analysis is capable of differentiating the training effects on novices’ learning and performance, with higher scores observed for those exposed to more training (Supplmement 1). However, most of those studies considered novices and suturing as the training task in simulation settings. It is worth mentioning that in this work, one expert was recruited following the procedure implemented in [5], which included 1 expert and 16 novices.

### Limitations

It considered only 1 basic laparoscopic task, so the conclusions should be interpreted cautiously and applied specifically to the peg transfer task . One another limitation is the inclusion of only 1 expert participant, which prevented statistical comparisons with the novice and intermediate groups. Instead, the expert’s performance was used as a reference to contextualize the differences between the novice and intermediate groups.

### Suggestions

It would be beneficial in the future to include testing of other Fundamentals of Laparoscopic Surgery tasks. Furthermore, higher-fidelity tasks can be considered for future research to introduce new conditions for laparoscopic training that simulate real situations in current surgical practice. Such tasks would be great additions to newly developed advanced laparoscopic tasks and would complement an advanced laparoscopic skills curriculum.

### Generalizability

This work presented Fundamentals of Laparoscopic Surgery for the first time in Jordan. However, the study was conducted at a single institution, which may limit its generalizability. Nonetheless, the integration of motion analysis scoring in simulation settings is straightforward, inexpensive, and reliable, with little guidance required, leaving no barrier for replication at other institutions.

### Conclusion

In this work, motion analysis of surgical instruments was presented to assess the performance of interns while performing a peg transfer task with uniform coloring in simulation-based settings. The motion analysis scoring was validated and correlated well with one of the most common methods for assessing minimally invasive surgery in a lab setting, GOALS. Both assessment methods effectively differentiated novices from intermediates.

## Figures and Tables

**Figure f8-jeehp-22-10:**
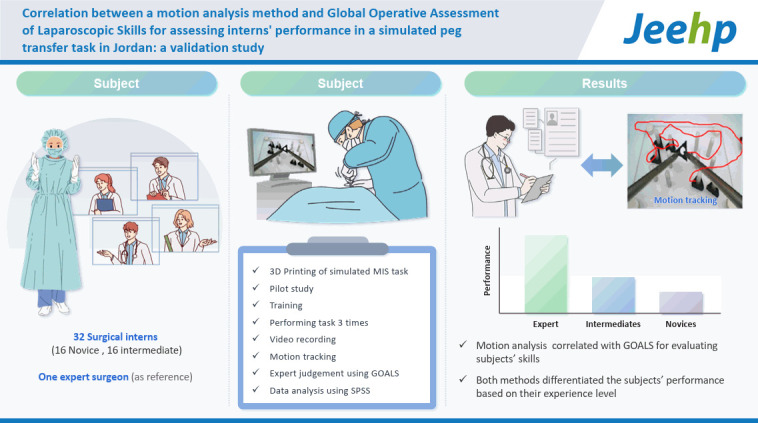


**Table 1.** Assessment methods, metrics, and definitions

	Definition
Motion analysis metrics	
Time	Minutes
Error	Number of dropped triangles per trial
Tremor	SD of the signal data minus data line of good fit
Extreme movement	SD of the data for line of good fit minus best fit
Overall pattern of movement	SD of the signal data
GOALS metrics^a)^	
Dexterity	Assesses the use of both hands in a complementary and optimal manner
Depth perception	Assesses the quality of movement toward the target accurately without missing it, swinging or overshooting
Efficiency	Assesses the number of wasted moves, grasps without leaving the scene
Autonomy	Assesses the ability to complete task without guidance
Operation flow	Assesses the ability to perform procedure with an appropriate pace and with planned course

SD, standard deviation.

a) GOALS (Global Operative Assessment of Laparoscopic Skills) metrics are assessed by raters on a scale from 1 to 5, with (1) being worst and (5) being the best.

**Table 2.** Participants demographics

Characteristic	Value
Gender	
Male	20 (62.5)
Female	12 (37.5)
Hand-dominance	
Right-handed	29 (90.6)
Left-handed	3 (9.4)
Vision	
20/20 (with or without corrective lenses)	32 (100.0)
Less than 20/20	0
Age (yr)	
Novices	24.5±5.5
Intermediates	29±3.2
Experience	
Novices	
No. of operations attended in general^a)^	18±20.8
No. of MIS operations attended^a)^	0±0
Intermediates	
No. of operations attended in general^a)^	48±2.8
No. of the MIS operations attended^a)^	6±7.3

Values are presented as number (%) or mean±standard deviation. Both intermediates and novices had the same number of females and males. Two left handed participants were intermediates and 1 novice. MIS, minimally invasive surgery.

a) Attended without participation.

## Footnotes

**Data availability**
Data files are available from Harvard Dataverse: https://doi.org/10.7910/DVN/TY8DQB Dataset 1. Cleaned quantitative research data which include all anonymized data for the study participants used for creating the study Figs. 2–7.

## Supplementary materials

Supplementary files are available from Harvard Dataverse: https://doi.org/10.7910/DVN/TY8DQB

Supplement 1. Summary table of additional referencing reinforcing research.

Supplement 2. Novice motion analysis raw data.

Supplement 3. Intermediate motion analysis raw data.

Supplement 4. Pseudo R-codes.

Supplement 5. Data for measurement of performance with motion analysis method for assessing interns’ performance in a simulated peg transfer task in Jordan.

Supplement 6. Data for measurement of performance with GOALS for assessing interns’ performance in a simulated peg transfer task in Jordan.

Supplement 7. Data for correlation between motion analysis and GOALS for assessing interns’ performance in a simulated peg transfer task in Jordan.

Supplement 8. Audio recording of the abstract.
