# Supplementary material for: Correlation between a motion analysis method and Global Operative Assessment of Laparoscopic Skills for assessing interns’ performance in a simulated peg transfer task in Jordan: a validation study
Source: J Educ Eval Health Prof. 2025 Mar 6;22:10. doi: 10.3352/jeehp.2025.22.10 (PMC12012728; doi:10.3352/jeehp.2025.22.10)
Supplement: Supplementary file 5 — Supplement 4. Pseudo R-codes. [file jeehp-22-10-suppl4.pdf]

Pseudo R-Code for extracting Tremor Score

```
signal_data <- c (Novice Motion Analysis Raw Data)

linear_model <- lm(signal_data ~ x_values)

fitted_values <- predict(linear_model)

residuals <- signal_data - fitted_values

sd_residuals <- sd(residuals)

print(sd_residuals)
```

Pseudo R-Code for extracting Extreme Movement Score

```
data <- c(Novice Motion Analysis Raw Data)

linear_model <- lm(data ~ x_values)

best_fit <- predict(linear_model)

residuals <- data - best_fit

sd_residuals <- sd(residuals)

print(sd_residuals)
```

Pseudo R-Code for extracting Movement Pattren Score

```
signal_data <- c(Novice Motion Analysis Raw Data)

sd_signal <- sd(signal_data)

print(sd_signal)
```

Note: The same codes can be used for Intermediates and Expert data
